# Inhibiting TLR4 signaling by linarin for preventing inflammatory response in osteoarthritis

**DOI:** 10.18632/aging.202469

**Published:** 2021-02-01

**Authors:** Weihui Qi, Yanlin Chen, Shuaibo Sun, Xinxian Xu, Jingdi Zhan, Zijian Yan, Ping Shang, Xiaoyun Pan, Haixiao Liu

**Affiliations:** 1 Department of Orthopedic Surgery, The Second Affiliated Hospital and Yuying Children’s Hospital of Wenzhou Medical University, Wenzhou 325027, China; 2 Department of Rehabilitation, The Second Affiliated Hospital and Yuying Children’s Hospital of Wenzhou Medical University, Wenzhou 325027, China

**Keywords:** linarin, TLR4, MD-2, chondrocyte, osteoarthritis

## Abstract

Osteoarthritis (OA) is one of the most common degenerative diseases, ultimately leading to long-term joint pain and severe articular malformation. Controlling local chronic inflammation is a crucial strategy for delaying OA development. Linarin is a natural flavonoid glycoside that is widely available in Compositae, Chrysanthemum indicum and Dendrocalamus and processes protective effects in several animal models. The purpose of our work was to study the protective effect of Linarin for OA. Cellular experiments data showed that Linarin suppressed lipopolysaccharide (LPS)-caused the overproduction of nitric oxide (NO), prostaglandin E2 (PGE2), interleukin-6 (IL-6) and tumour necrosis factor-alpha (TNF-α) in chondrocyte. In addition, LPS-stimulated expression of cyclooxygenase-2 (COX-2) and inducible nitric oxide nitrate (iNOS) was decreased by Linarin pre-treatment. Together, Linarin prevented the catabiosis of extracellular matrix caused by LPS. For mechanism, Linarin inhibited the formation of Toll-like receptor 4 (TLR4) / myeloid differentiation protein-2 (MD-2) dipolymer complex and subsequently intervened NF-κB activation. Our mouse DMM model further clarified the protection of Linarin *in vivo*. In summary, our results suggested that Linarin may be a potential effective agent for OA.

## INTRODUCTION

Osteoarthritis (OA) is one of the most common joint diseases characterized by synovial inflammation, osteophyte formation, degeneration of cartilage and subchondral bone changes in histopathology, but lacks satisfactory therapy. [1–3] Inflammatory drugs (NASIDs) for OA could temporarily relieve the clinical symptoms but induce several adverse reactions. [4] Total knee arthroplasty is limited to patients with advanced OA, and the cost of surgery is huge. The pathogeny of OA is unclear and complicated. However, it has been found to be related to obesity, metabolic abnormalities, aging and osteoporosis. [5–8] It is widely accepted that multiple inflammatory responses participate in OA process. [9, 10] Lipopolysaccharides (LPS) could lead to low-grade inflammation and be involved in some clinical diseases, including cardiac dysfunction, atherosclerosis and diabetes. [11] It is not clear what the key role of LPS is in the development of the mentioned diseases. Huang et al. found that LPS and LPS binding protein (LBP) levels were increased in both synovial fluid (SF) and serum of individuals with OA compared to the normal group. [11, 12] Therefore, LPS could serve as an effective stimulus in the study of OA *in vitro*. [13, 14].

Toll-like receptors (TLRs) recognize different pathogen-related molecular patterns and induce various pathophysiological processes such as immune cell regulation, inflammation, survival and proliferation. [15–17] In chondrocytes, TLR4 is the most important TLR contributing to OA development. [18] LPS recognizes TLR4 receptor to trigger a series of subsequent inflammatory reactions. [14] This process requires the involvement of myeloid differentiation protein-2 (MD-2), the extracellular domain of TLR4, which could specifically bind to LPS and form LPS / TLR4 / MD-2 complexes. [19, 20] These newly synthesized complexes bind downstream proteins such as myeloid differentiation factor 88 (MyD88), tumor necrosis factor receptor-related factor 6 (TRAF6), and interleukin-1-receptor-associated kinases (IRAKs) to induce activation of signal transduction pathways including NF-κB and MAPK. [21] This complex multi-stage reaction leads to inflammation and catabolic occurrence. Therefore, inhibiting the production of LPS/TLR4/MD-2 complex may be promising for OA therapy. [22].

Linarin, also known as Robinin, is a natural flavonoid glycoside widely existing in Compositae, Chrysanthemum indicum, Dendrocalamus and other plants. [23] Modern pharmacological studies have demonstrated that Linarin has anti-inflammatory and analgesic effects and can inhibit the expression of inflammatory mediators such as interleukin (IL) and tumor necrosis factor (TNF). [24, 25] Previous studies have shown that Linarin suppresses LPS-caused lung injury by inhibiting inflammation and oxidative stress via regulating TXNIP/NLRP3 and NF-κB signaling. [26] In addition, Linarin’s anti-inflammatory role has been discovered in acute liver failure model caused by LPS and D-galactosamine. [27] However, the protection and underlying mechanism of Linarin for OA have not been studied. The purpose of this study was to investigate the anti-inflammatory effect and mechanism of Linarin on human OA chondrocytes induced by LPS and the protective effect of Linarin against cartilage degeneration in mice OA model.

## RESULTS

### Effect of linarin on human OA chondrocyte viability

**Figure 1:**
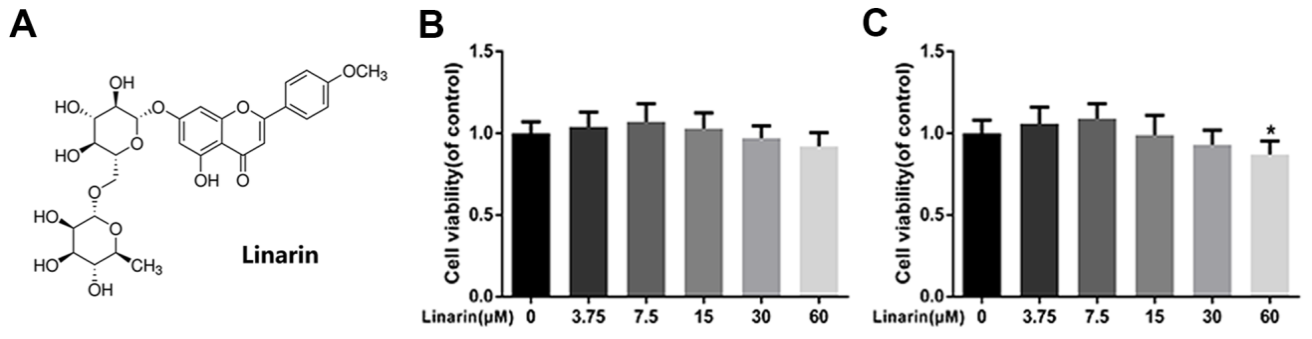
**Effect of Linarin on human chondrocytes viability.** (**A**) Chemical structure of Linarin. (**B**, **C**) Cytotoxicity of Linarin on chondrocytes at 24h and 48h. The data in the figures represent the averages ± S.D. Significant differences among different groups are indicated as *P < 0.05 vs. control group, n=5.

Figure 1A shows the chemical structure of Linarin. The cytotoxicity effect of the Linarin on chondrocytes was detected by CCK8 kit with increasing concentration (0, 3.75, 7.5, 15, 30, 60 μM) at 24 and 48 hours (Figure 1B, 1C). After 48 hours treatment, the viability of cells in 60 μM group was significantly decreased relative to 0 μM group. Therefore, the dose used in the following experiments were 7.5, 15 and 30 μM.

### Effects of linarin on the expression of COX-2, iNOS, TNF-α, NO, IL-6 and PGE2 in human OA chondrocytes induced by LPS

**Figure 2:**
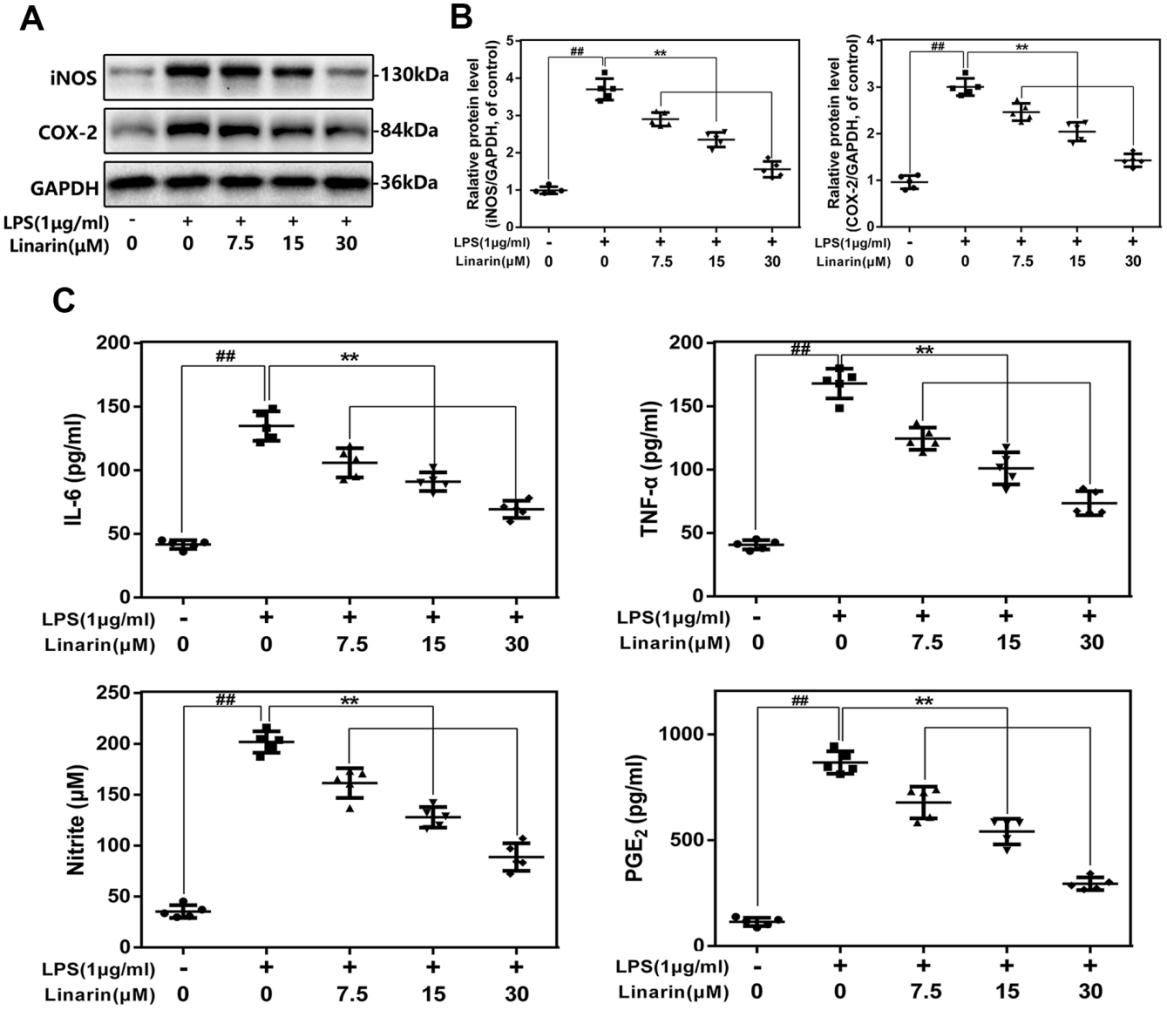
**Influence of Linarin in LPS-induced inflammatory reaction in human chondrocytes.** (**A**, **B**) iNOS and COX-2 protein level in chondrocytes measured by western blot. (**C**) Effect of Linarin on LPS-exposed IL-6, TNF-α, PGE2, and NO production in human chondrocytes. The data in the figures represent the averages ± S.D. Significant differences among different groups are indicated as ^##^*P* < 0.01, vs. control group; **P < 0.01 vs. LPS alone treatment group, n=5.

We performed western blot to detect the expression of COX-2 and iNOS at protein levels. As shown in Figure 2A, 2B, Linarin inhibits the LPS-caused COX-2 and iNOS overexpression in a drug-dependent form (7.5, 15 and 30 μM). The external generation level of TNF-α, NO, IL-6 and PGE2 are increased after the stimulation of LPS, while Linarin pretreatment reversed these changes (Figure 2C).

### Effects of linarin on LPS-induced extracellular matrix (ECM) catabiosis in human OA chondrocytes

**Figure 3:**
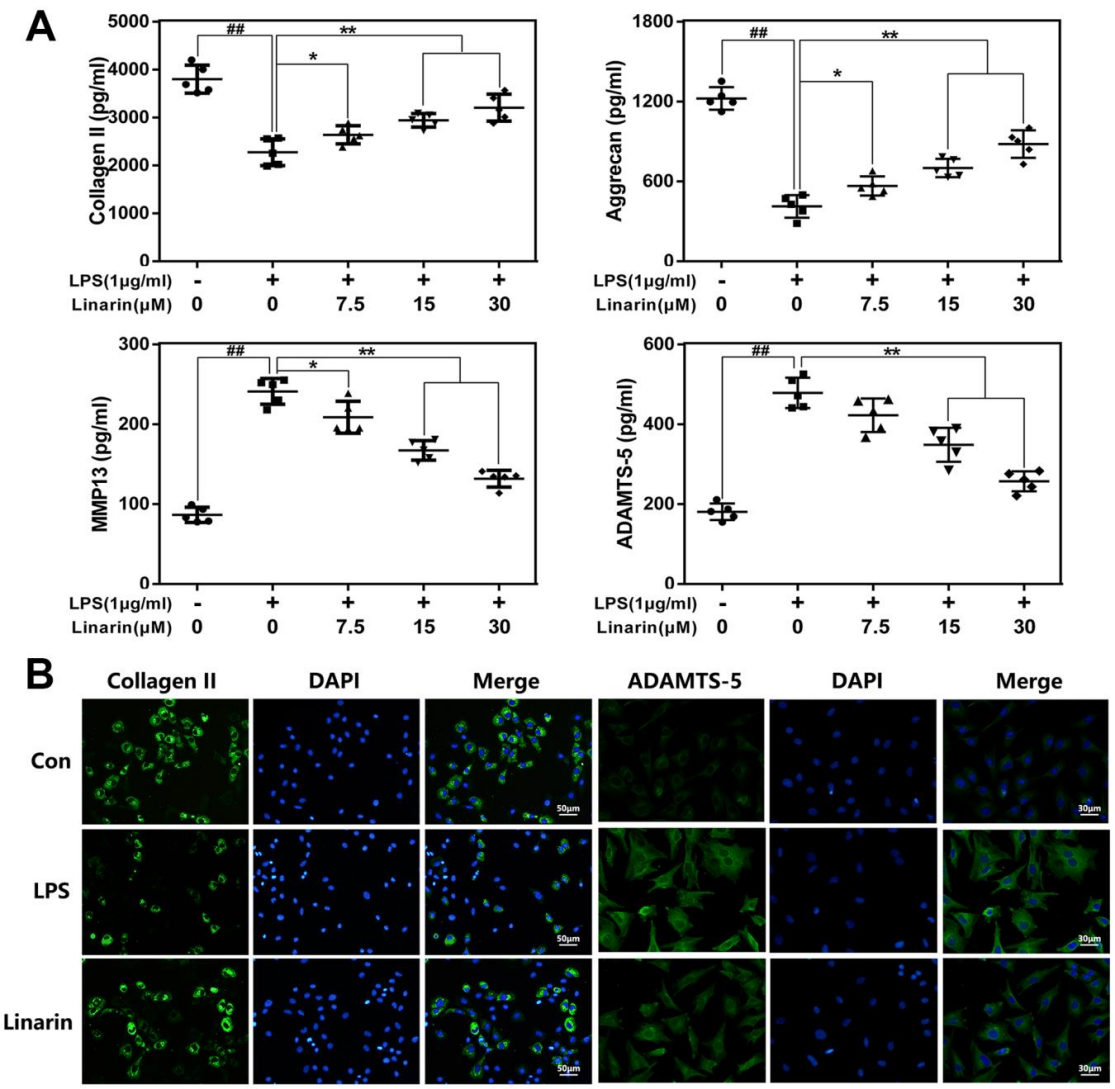
**Influence of Linarin in LPS-medicated ECM degeneration in human chondrocytes.** (**A**) The level of Collagen II, aggrecan, MMP13 and ADAMTS-5 in chondrocytes treated as above were visualized by ELISA. (**B**) The representative collagen II and MMP13 was detected by the immunofluorescence combined with DAPI staining for nuclei (scale bar: 50μm or 30μm). The data in the figures represent the averages ± S.D. Significant differences among different groups are indicated as ^##^*P* < 0.01, vs. control group; *P < 0.05, **P < 0.01 vs. LPS alone treatment group, n=5.

Aggrecan and collagen II are essential ingredients of ECM in maintaining chondrocyte function and survival. We assessed the role of Linarin in ECM degradation in chondrocytes exposed to LPS. As shown in Figure 3A, ELISA results illustrated that Linarin pretreatment enhances the expression of aggrecan and collagen II and inhibits the expression of ADAMTS-5 and MMP13 relative to the single LPS group. In addition, immunofluorescence result about collagen II and MMP13 are consistent with ELISA assay (Figure 3B). These results showed that Linarin can reduce ECM degradation. Of note, Linarin treatment alone do not affect iNOS, COX2, Collagen II and ADAMTS5 proteins expression (Supplementary Figure 1).

### Effects of linarin on LPS-induced activation of NF-κB in human OA chondrocytes

**Figure 4:**
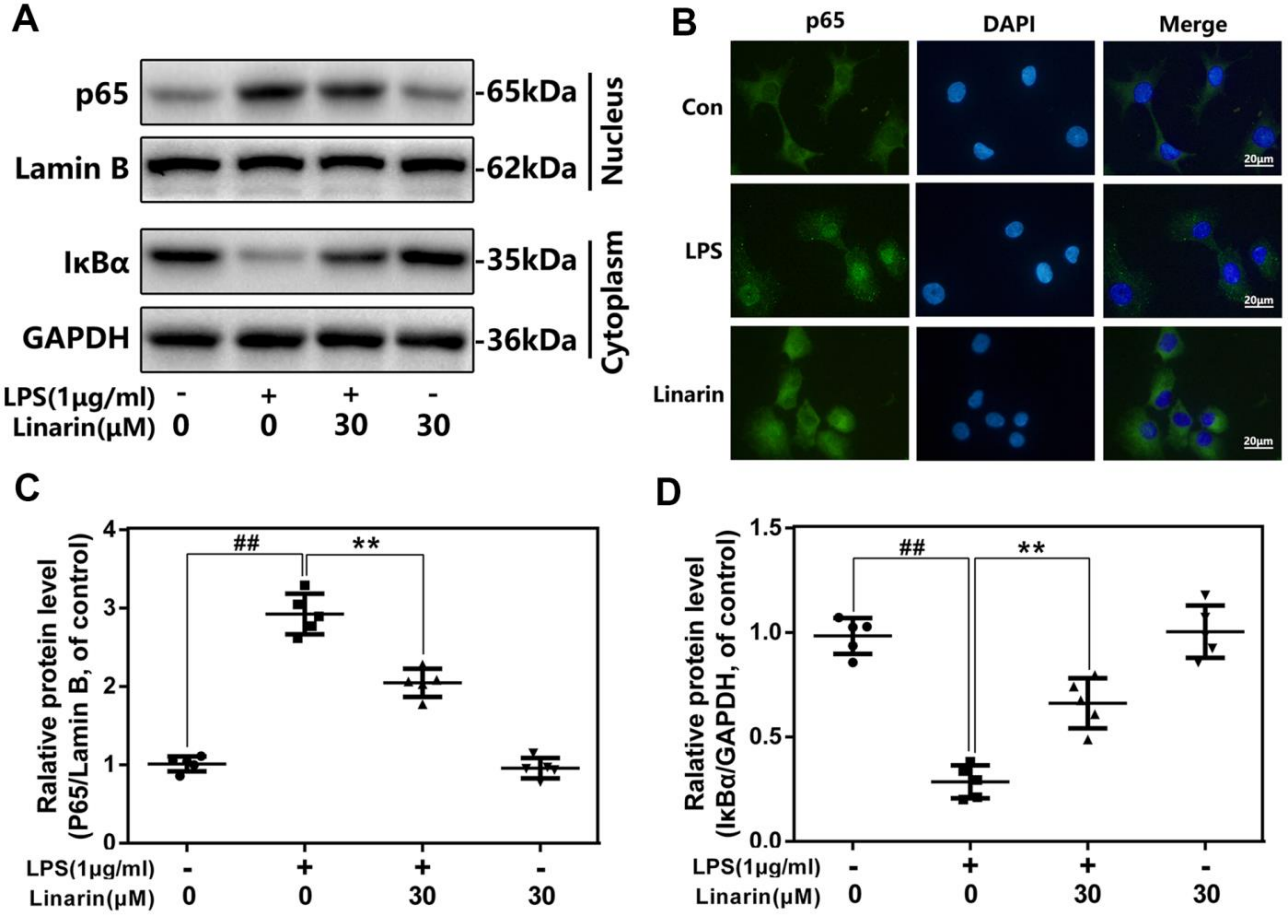
**Influence of Linarin in LPS-medicated activation of NF-κB pathway in human chondrocytes.** (**A**, **C**, **D**) The protein expressions of IκBα in cytoplasm and p65 in nuclear in chondrocytes treated as above were detected by western blot. (**B**) The nuclei translocation of p65 was detected by the immunofluorescence combined with DAPI staining for nuclei (scale bar: 20 μm). The data in the figures represent the averages ± S.D. Significant differences among different groups are indicated as ^##^*P* < 0.01, vs. control group; **P < 0.01 vs. LPS alone treatment group, n=5.

To investigate mechanism of Linarin in inflammation, western blot analysis and immunofluorescence were performed to detect the activity of NF-κB signaling. LPS stimulated the degradation of IκBα in the cytosol and activated p65 transfer into nucleus. Nevertheless, these phenomena were markedly inhibited by the 30 μM dose of Linarin pretreatment. There was no significant change in NF-κB activity in the Linarin treatment group alone (Figure 4A, 4C, 4D). Furthermore, immunofluorescence showed that most of the p65 protein was presented in the cytosol, but LPS stimulation promoted p65 transfer into the nucleus (Figure 4B). Linarin pretreatment partially decreased p65 transfer. In summary, these data showed that Linarin blocks the activation of NF-κB in human OA chondrocytes stimulated by LPS.

### Effect of linarin on the interaction of TLR-4 and MD-2 in human OA chondrocytes induced by LPS

**Figure 5:**
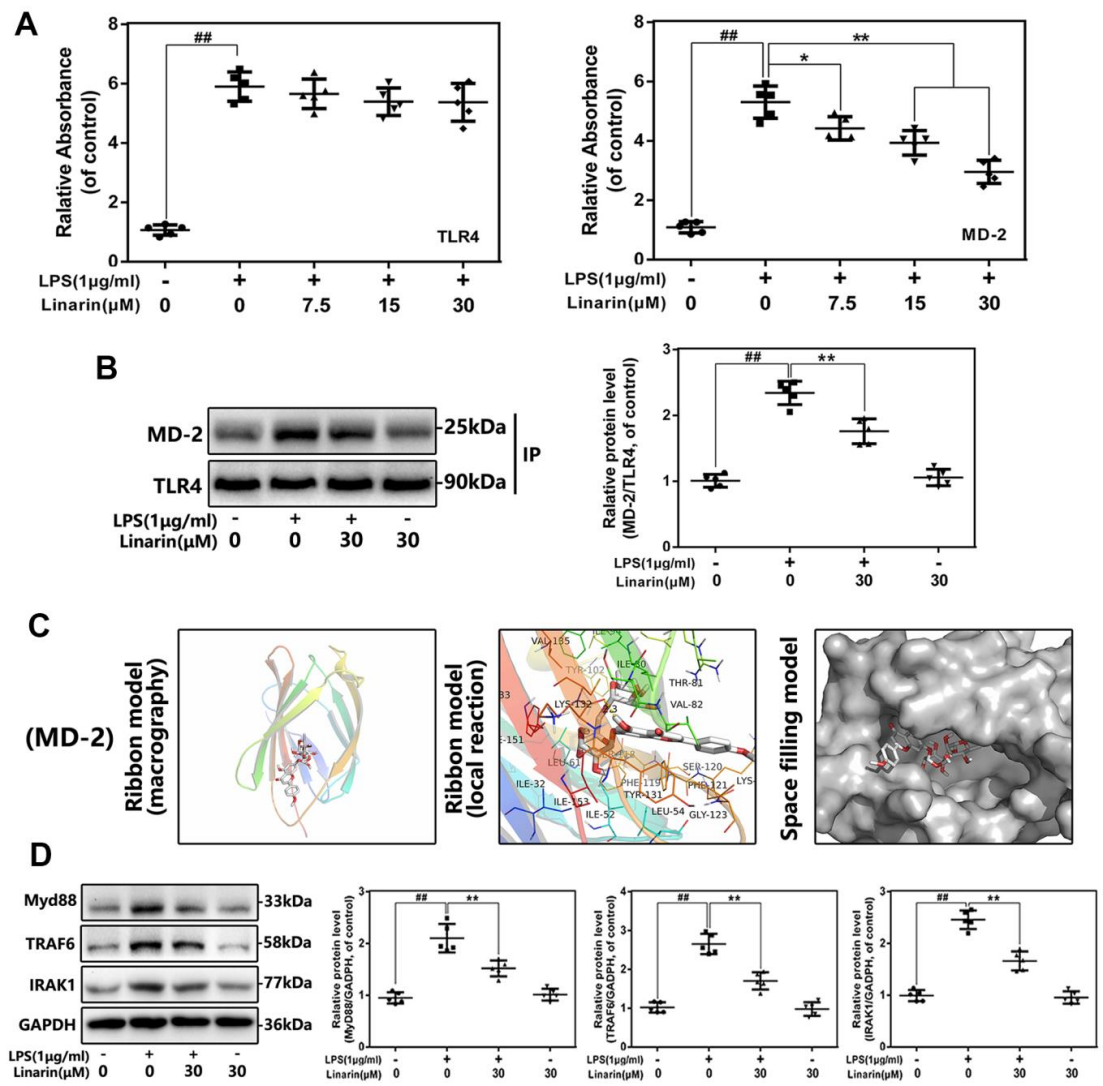
**Influence of Linarin on LPS-induced TLR4/MD-2 signaling activation.** (**A**) The binding of biotin labeled LPS to rhMD-2 and rhTLR4 was examined by competitive ELISA. (**B**)The complexes of TLR4-MD-2 in chondrocytes treated as above were detected by immunoprecipitation. (**C**) Linarin was docked with the MD-2 structure. Docking studies were performed as described in Materials and methods. The protein residues are shown in a ribbon model. The proposed binding pose of Linarin shows interactions with LYS-132. The space filling models show the binding of Linarin in the inhibitory binding pockets. (**D**) The protein expressions of MyD88, IRAK-1 and TRAF-6 in chondrocytes treated as above were detected by western blot. The data in the figures represent the averages ± S.D. Significant differences among different groups are indicated as ^##^*P* < 0.01, vs. control group; **P < 0.01 vs. LPS alone treatment group, n=5.

TLR4 is an upstream molecule of NF-κB signaling that has been extensively studied. TLR4 agonists (such as LPS) cannot be directly connected to the receptor, but need to be mediated by the adaptor protein MD-2. To examine whether Linarin inhibits the interaction between LPS and TLR4 / MD-2, the competitive ELISA and co-immunoprecipitation were performed. Competitive ELISA showed that Linarin reduced the connection between LPS and rhMD-2 but had no effect on rhTLR4 (Figure 5A). And co-immunoprecipitation results support this phenomenon. LPS promotes the interaction between MD-2 and TLR4, while Linarin inhibits this phenomenon. However, Linarin did not interfere the formation of TLR4 / MD-2 complex when treatment alone (Figure 5B). These results show that Linarin possesses a competitive LPS site on the TLR4 / MD-2 complex. Next, we conducted molecular docking analysis. As show in Figure 5C, Linarin exerts high affinity for MD-2, which was -7.3 kcal mol^-1^. The ribbon model demonstrates macrography and local reactions between Linarin and protein residues, and a hydrogen bonds are formed between Linarin and the LYS-132 residue of MD-2 through the ribbon model. The space filling model indicates that the inhibitory region of the corresponding protein completely encapsulates the molecular structure of Linarin. These data indicated that Linarin could intervene the formation of TLR4 / MD-2 complex stimulated by LPS (Figure 5C).

### Effect of linarin on activation of toll adapters in LPS-induced human OA chondrocytes

Several intracellular adapters, including TRAF-6, IRAK-1 and MyD88, are participated in LPS-induced TLR4 signaling activation, which is critical for NF-κB-mediated inflammation. The protein levels of TRAF-6, IRAK-1, and MyD88 have been investigated by western blot analysis, and the effect of Linarin on these cascading signaling in human chondrocytes has been confirmed. LPS treatment promotes the expression of TRAF-6, IRAK-1 and MyD88, but Linarin pretreatment reverses these phenomena. These data demonstrate that the Linarin's anti-inflammatory effect may be mediated by the TLR4 / MyD88 axis (Figure 5D).

### Linarin ameliorates OA development in a mouse model of DMM

**Figure 6:**
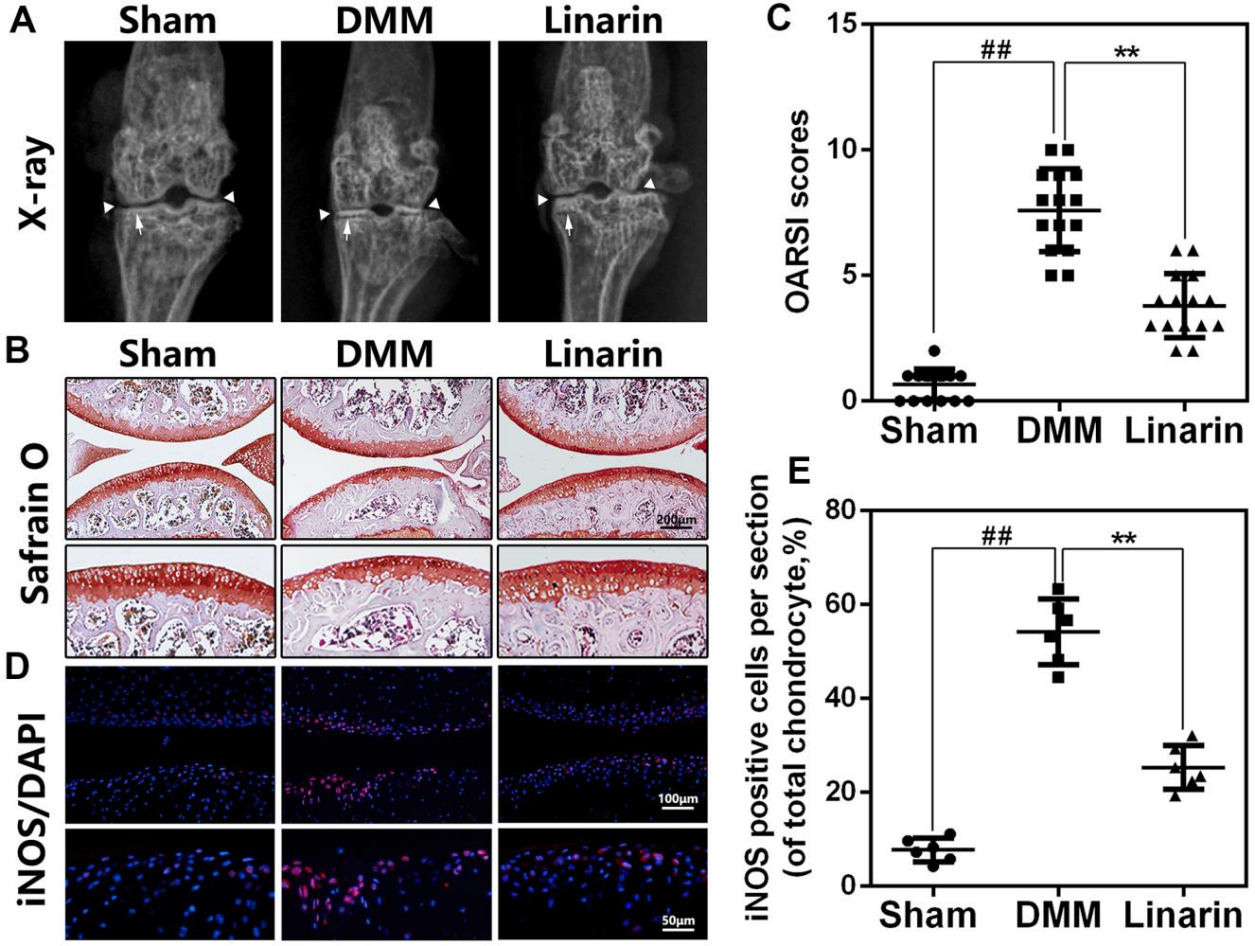
**Linarin attenuates OA development *in vivo*.** (**A**). Digital X-ray image of mouse knee joints from different experimental groups. Narrowing of joint space was found in both OA and treatment group (white triangles), the calcification of cartilage surface was obviously shown in OA group (white arrows). (**B**). Representative S-O staining of cartilage from different experimental groups at 8 weeks post-surgery (scale bar: 200 μm). (**C**). Diagrams showed the OARSI scores of cartilage. (**D**, **E**) The immunofluorescence staining of iNOS in mouse knee articular cartilage (scale bar: 20 μm). The data in the figures represent the averages ± S.D. Significant differences among different groups are indicated as ^##^*P*<0.01, **P<0.01.

In order to demonstrate protection of Linarin *in vivo*, DMM mouse model was performed by surgery to simulate the development of OA. Later, 30 mg / kg of Linarin were taken orally once a day for 8 weeks with some mice suffering DMM surgery. By X-ray results, we obviously found a narrower joint area and calcification of cartilage surfaces in the OA group compared to the sham operations group. Nevertheless, the calcification of cartilage surfaces in the DMM+Linarin group is weak, even with a wider joint area (Figure 6A). Safranin O staining was conducted to estimate the cartilage histological analysis. Compare to the sham group, the cartilage surface in the DMM group was rougher, with lighter red, chondrocyte loss, and a higher OARSI score (Figure 6B, 6C). But in DMM+Linarin group, these pathological changes were significantly improved and the OARSI score was significantly reduced (Figure 6C). Additionally, Linarin treatment inhibited DMM-caused iNOS expression in mouse cartilage, indicating that the inflammatory response in OA chondrocytes was suppressed by Linarin (Figure 6D, 6E).

## DISCUSSION

Linarin is a kind of natural flavone glycosides widely existing in Compositae, Chrysanthemum indicum, Dendrocalamus officinalis and other plants. [23] Previous studies have shown that Linarin have multiple pharmacological activities especially anti-inflammatory. [24, 26, 28] However, the inflammatory mechanism of Linarin in OA still needs to be studied.

Numerous studies demonstrated that inflammatory mediators play a vital role in the development of OA. [9, 29] In these mediators, iNOS promotes MMPs synthesis and secretion by catalyzing NO production, subsequently accelerate ECM degradation. [30, 31] As an inflammation mediator, prostaglandin E2 (PGE2) is the degradation product of arachidonic acid mediated by LPS via COX-2 and is related to the overexpression of ADAMTs and MMPs. [32, 33] Additionally IL-6 and TNF-α are involved in OA progress. [30, 32] In our study, Linarin pretreatment significantly inhibited the LPS-induced abnormal increase of PGE2 and NO in human OA chondrocytes and the upregulation of COX-2 and iNOS. The results found with the TNF-α and IL-6 were similar. For ECM metabolism, Linarin reversed LPS caused overproduction of MMP13 and ADAMTS-5 and degeneration of collagen II and aggrecan. These data indicate that Linarin has a potential therapeutic role in preventing OA development.

For the specific mechanism of Linarin, we found that the activation of NF-κB signaling stimulated by LPS was inhibited by Linarin. It is consistent with Sun et al., who reported that Linarin prevents acute lung injury caused by LPS by inhibiting the NF-κB pathway. [26] But, we believe that there are still some upstream targets at work, not directly intervene NF-κB pathway. As a conserved transmembrane protein, TLR4 receptor has been demonstrated to dominate multiple inflammatory signal paths in OA development. [21] For activation of TLR4-related signals, LPS acts as a pathogen-related molecular patterns (PAMPs) and can be recognized and bound with TLR4-MD-2 heterodimer. [34] Briefly, LPS binds two identical TLR4-MD2 structures to product two symmetrically-arranged TLR4–MD-2–LPS complex. [19] For the specific structure of the complex, LPS has six lipid chains, five of which are wrapped in the MD-2's hydrophobic pocket, and the remaining one is exposed to the periphery and can form a hydrophobic bond with TLR4. [35] In our study, competitive ELISA showed that Linarin could prevent LPS from binding to MD-2, but does not show significant effect on TLR-4. Co-immunoprecipitation showed that Linarin could significantly weaken the interaction between TLR4 and MD-2 induced by LPS, and Linarin molecule specifically linked with the LYS-132 residue of MD-2 through a hydrogen bond was proved by docking analysis. In LPS-mediated mouse liver injury model, Linarin treatment inhibited inflammatory factors production and secretion including IFN-γ, IL-6 and TNF-α via the intervene of TLR4 / IRAK pathway, which are roughly the same as our results. [27] With the formation of TLR4-MD-2–LPS complex, the intracellular domain of TLR4 undergoes polymerization changes to form the Toll/IL-1R homology (TIR) domain, which subsequently recruit MyD88, IRAK1 and TRAF6 to form a complex to activate downstream signals. [35–37] Our western blot results demonstrated that the LPS-induced overexpression of IRAK-1, TRAF-6 and MyD88 in chondrocyte was significantly suppressed by Linarin pretreatment.

For *in vivo* study, we constructed mouse knee joint DMM model to evaluate the protective effect of Linarin for OA. The knee joint showed cartilage calcification, erosion and subchondral changes in DMM group, accompanied with increased OARSI scores. Nevertheless, Linarin improved these phenomena and lowered the OARSI scores.

## CONCLUSIONS

**Figure 7:**
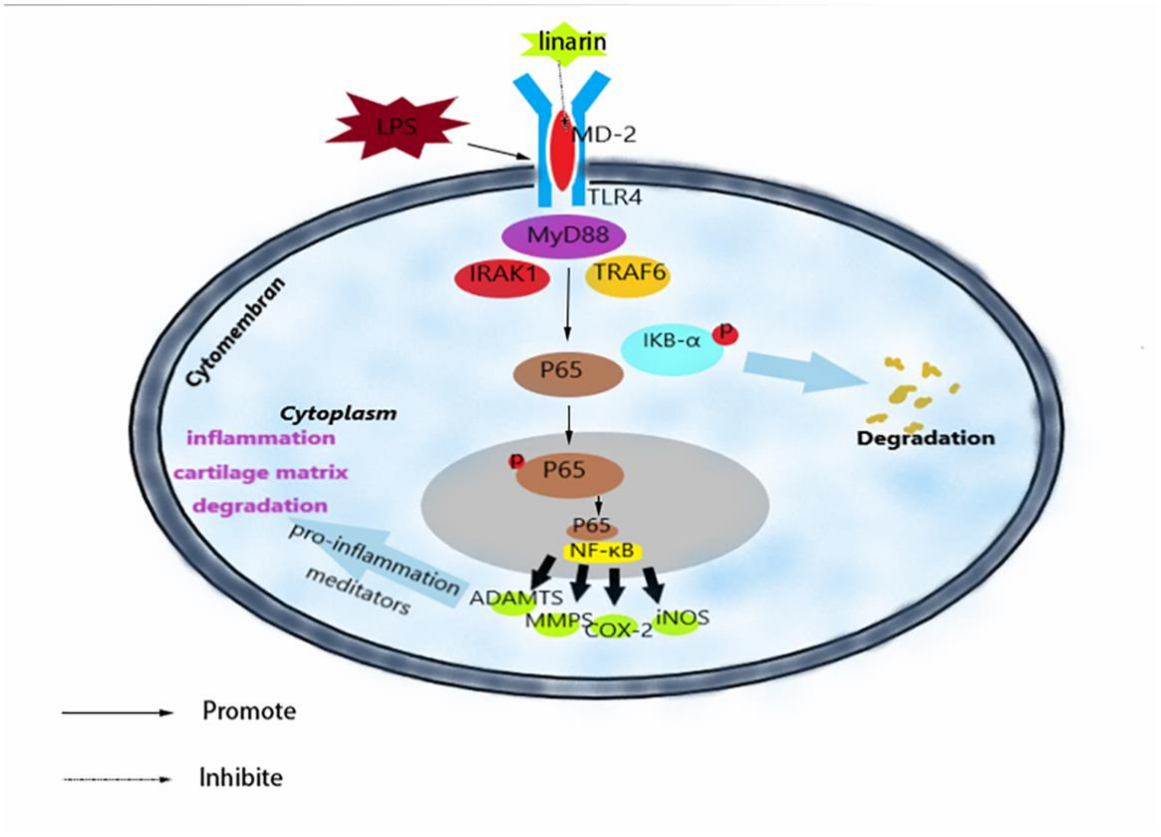
**Schematic illustration of the potential protection of Linarin in OA process.**

To summarize, we have shown that Linarin inhibits LPS-medicated inflammation via inhibiting TLR4-NF-κB signaling axis in human chondrocytes (Figure 7). In addition, oral administration of Linarin suppressed OA development in mouse DMM model induced by surgery. Our research data confirmed the potential therapeutic role of Linarin in the progression of OA.

## MATERIALS AND METHODS

### Chemicals and reagents

Linarin (purity>98%) was brought from BOC sciences (New York, NY, USA). The antibodies against MD-2 was obtained from eBioscience (eBioscience, San Diego, CA, USA) and Lamin B1, GADPH, TRAF6, TLR4, MyD88 were from Abcam (Cambridge, UK) and IκBα, p65 and COX-2 were from Cell Signaling Technology (Danvers, MA, USA). LPS labeled Biotin (Biotin-LPS) purchased from Invitrogen (San Diego, CA, USA). AlexaFluor®594 and AlexaFluor® 488, which have IgG (H + L) goat bargain antibodies labeled from Jackson Immunoresearch (West Grove, PA, USA). Recombinant TLR4 and MD2 human proteins (rhTLR4 and rhMD2) were obtained from R&D Systems (Minneapolis, MN, USA).

### Primary human chondrocyte culture

According to the guidelines of Helsinki and Tokyo, and the rules of the Medical Ethics Committee of the Second Hospital of Wenzhou Medical University, we collected human articular cartilage tissue from 5 patients (age 55-67 years, 3 men, 2 women) who underwent TKA (Total Knee Arthroplasty) for the OA at the Second Affiliated Hospital of Wenzhou Medical University. The study obtained informed consent from all patients. Cartilage tissue was cut small pieces (about 1*1*1 mm^3^) and washed 3 times with PBS. Then the tissue fragments were digested with trypsin-EDTA 0.25% for 30 minutes at 37° C. The hydrolysis solution was replaced by collagenase II (0.2%) at 37° C for 4 hours, followed by centrifugal at 1000 rpm for 5 minutes and discard the supernatant. The chondrocytes were cultured in a 6-hole plate at a density of 2 x 10^5^ cells per milliliter. Cells were transferred when combined at 80 to 90% by 0.25% TRYPSIN-EDTA solution.

### Animal OA models

45 8-week-old C57BL / 6 wild male species (WT) mice were obtained from the Animal Center of the Chinese Academy of Sciences in Shanghai. All of the experimental procedures involving the animal use and care were approved by the Animal Care and Use Committee of Wenzhou Medical University (wydw-2018-202) and abided by the guide for the care and use of laboratory animals of the national institutes of health. The destabilisation of the medial meniscus (DMM) as mentioned previously [38], incised the right knee of the mice with a patella tendon in the middle and the tendon between the medial meniscus and the tibial plateau was cut with a small surgical scissors. And in the sham group, only arthrotomy was performed without cutting the medial meniscotibial ligament.

### Experimental design

For the *in vitro* studies, the chondrocytes were treated with LPS (1 μg / mL) either alone or with various concentrations (7.5, 15, 30 μM) of the Linarin pretreatment. The LPS stimulation duration was 24 hours to check for functional changes such as inflammation and ECM degeneration and 2 hours to detect NF-κB and TLR4 signals activation. Of note, the high concentrations of lipopolysaccharides have been used to study the mechanism of cell death in previous studies, while low concentrations of lipopolysaccharides is more suitable for our study of inflammation. [11, 39] And previous studies have shown that doses of LPS lower than 1 μg/ml^−1^ do not significantly change cell viability. [22, 40] Therefore, the concentration of LPS was 1 μg/ml^−1^ in this study.

As for *in vivo* experiments, 45 mice were randomly divided into 3 groups (sham, DMM, DMM+Linarin), 15 mice in each group. After DMM surgery, we divided the mice into DMM group and DMM+Linarin group. The mice in DMM+Linarin group were treated with 30 mg/kg/day Linarin (10% Tween 80 in saline) by gavage. Meanwhile, the mice in DMM group were treated with an amount equal to 10% Tween 80 in saline. At 8 weeks after surgery, we sacrificed all mice and harvested cartilage tissue for imaging and histological studies.

### Cell viability

The viability of the cells was measured by CCK-8 in accordance with the manufacturer's protocol. In brief, cells were first cultured in 96 holes for 24 hours at a density of 5 x 10^3^ cells per hole and followed by additional 24 hour and 48 hours incubation with various concentrations (0, 3.75, 7.5, 15, 30, 60 μM) of Linarin, then added 100μl DMEM / F12 with 10 μl CCK8 solution added to each hole and curing for 4 hours at 37° C. Absorption of each hole was read by a spectrophotometer (Thermofisher) at 450 nm and all the experiments completed 5 times.

### ELISA analyze

The concentrations of TNF-α, PGE2, collagen II, IL-6, aggrecan, MMP13 and ADAMTS-5 in cultured cells were mainly available in Commercially measured by the ELISA series (R & D Systems, Minneapolis, MN) in accordance with the manufacturer's protocol. All experiments were repeated at least 5 times.

### Protein extraction and western blotting

Chondrocytes proteins were extracted using the RIPA lysis buffer, and using nuclear protein and cytoplasm extraction kits (Beotomy, Shanghai, China) to extract the corresponding proteins. Vibrated on ice, then centrifuged at 12,000 rpm and 4° C for 30 minutes. The protein concentration was then determined using a BCA protein test kit (Beyotime, Shanghai, China). The same amount of protein (40 ng) was separated using sodium dodecyl sulfate-poly acrylamide gel electrophoresis (SDS PAGE) and the protein was transferred to the membrane. Subsequently, the membrane was blocked with 5% nonfat milk for at room temperature 2 hours after washed by 0.1% Tween-20 in the TRIS buffer (TBST). The drops were incubated with primary antibodies overnight at 4° C. TLR4 (1: 500), MD-2 (1: 500), IκBα (1: 1000), COX-2 (1: 1,000), iNOS (1: 1,000), p65 (1: 1,000), IRAK1 (1: 500), TRAF6 (1: 1,000), MyD88 (1: 1,000), GADPH (1: 5000) and Lamin- B (1: 5000). The next day, the membrane was incubated with suitable secondary antibodies. The blots were observed by incubating with electrochemiluminescence plus reagent (Invitrogen), after washing with 3 times of TBST. Eventually, the intensity of these blots was quantitative analyzed using Image Lab software. 3.0 (Bio-Rad).

### MD-2 and TLR4 competitive ELISA assay

The non-cell ELISA competition assay is used to determine the ability of Linarin to involve in LPS binding to MD-2 and TLR4. Human TLR4 or MD-2 antibodies are coated on 96 holes (polystyrene holes) in 10mM Tris HCl buffer (pH 7.5). The dish is incubated at 4° C overnight, and the next day, sealed with 3% BSA (Bovine serum albumin) at room temperature for 1.5 hours after washed with PBST (polybutylene succinate-co-butylene terephthalate). Subsequently, 4 μg/ml of recombinant human Toll-like receptor 4 (rhTLR4) or recombinant human MD-2 (rhMD-2) is added and followed by additional 1.5 hours incubation at room temperature. Then, add biotin-LPS at room temperature with or without Linarin (7.5, 15, or 30 M). Streptavidin-conjugated peroxidase (Beyotime, Shanghai, China) was added at room temperature incubated for 1 hour. After using of tetramethylbenzidine substrate (eBioscience, San Diego, CA, USA), the activity of Horradio Oxidation was measured at 450 nanometers using spimax M5 disc reader (molecular device from Sunnyvale, CA, USA).

### Immunoprecipitation

After treatment, cell lysine was collected and incubated with antibody against TLR4 for 1 hour. Immune complexes are restored at 4° C overnight with G-sepharose beads. Sediment is washed with cold PBS for 4 times and then boiled in a buffer. Then, using the anti-MD-2 antibody, further detection of MD-2 levels was done using immunoblotting.

### Molecular modeling

The molecular structure of Linarin was produced by ChemBioDraw, and using ChemBio3D to imitate energy. Access the Protein Data Bank (https://www.rcsb.org/) to gain the crystal structure of the human MD2 / lipid IVa complex (PDB code 2E59). After being minimized with PyMoL (version 1.7.6), the lowest docking energy conformations were determined by default parameters. For protein and ligand analysis, we used the AutoDock tool (version 1.5.6). Otherwise, the AutoDock tool (version 1.5.6) provided pocket binding residue for ligand binding flexibility. With the help of UCSF PyMoL, finally the 3D viewer was created.

### X-ray imaging method

After 8 weeks of surgery, animals received an X-ray. Knee joint X-ray was produced by using the Digital X-ray machine (Kubtec Model XPERT.8; KUB Technologies Inc) at 160μA and 50 kV.

### Histological analysis

Knee samples were modified with 4% paraffin formaldehyde for 24 hours and then decalcified in 10% EDTA solution at 4° C for 4 weeks. Then the sample was dehydrated by gradient ethanol, embedded in paraffin. Finally, sample was sliced into 5um-sized pieces. And 10 slides each joint at every 50 μm were chosen and stained with Safranin O/Fast Green to evaluate the degeneration of articular cartilage.

Experienced histological researchers who were blinded to the experimental group evaluated cartilage destruction and assessed the medial femoral condyle and medial tibial plateau using the Osteoarthritis Research Society International (OARSI) scoring system as described previously. [38] Fifteen mice per group were selected for histomorphometric evaluation.

### Statistical analysis

The experiments were repeated at least 5 times. The SPSS 20.0 statistical software was used to display the experimental data as mean ± S.D. Data analysis was expressed as mean ± S.D, compared by one-way analysis of variance (ANOVA), and then Tukey test. *P* <0.05 was considered statistically significant.

### Data availability statement

The data used to support the findings of this study are available from the corresponding author upon request.

## Supplementary Material

Supplementary Figure 1
