# Supplementary material for: Inhibiting TLR4 signaling by linarin for preventing inflammatory response in osteoarthritis
Source: Aging (Albany NY). 2021 Feb 1;13(4):5369–82. doi: 10.18632/aging.202469 (PMC7950270; doi:10.18632/aging.202469)
Supplement: Supplementary Figure 1 [file aging-13-202469-s001.pdf]

SUPPLEMENTARY FIGURE

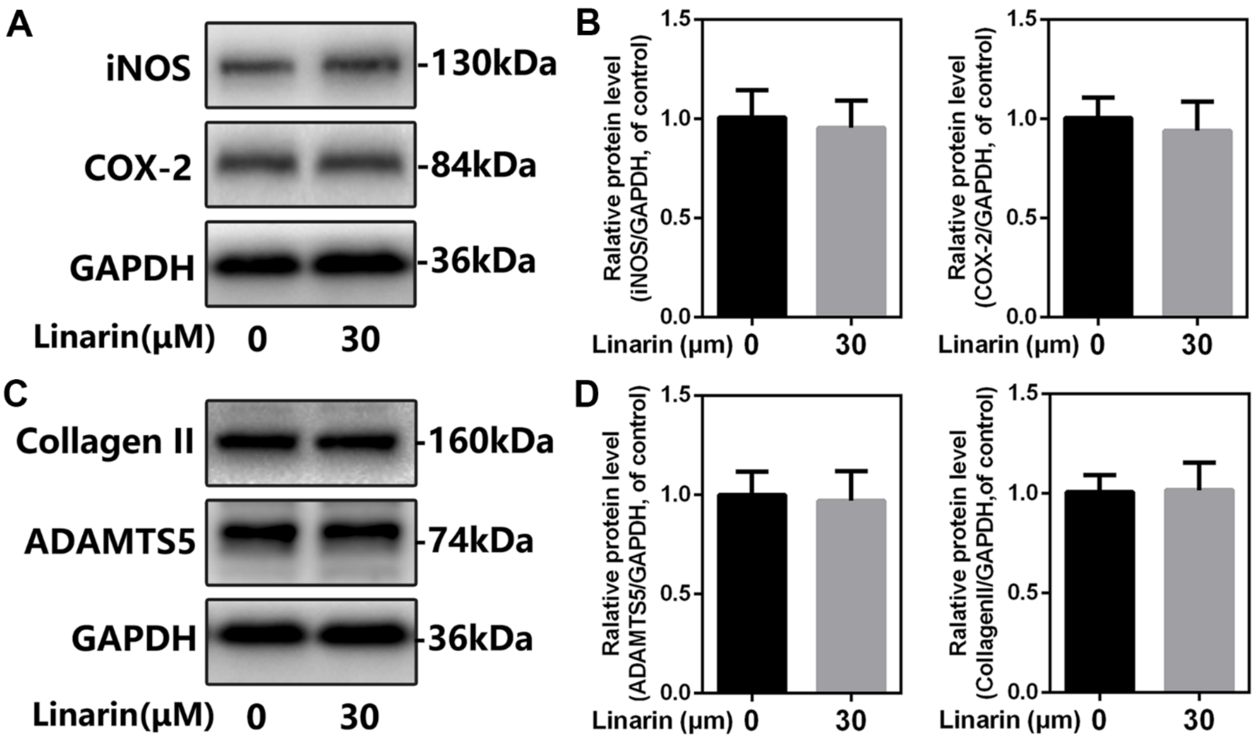

Supplementary Figure 1. Effect of Linarin alone treatment on chondrocyte inflammation and ECM protein. (**A**, **B**) iNOS and COX-2 protein level in chondrocytes measured by western blot. (**C**, **D**) Collagen II and ADAMTS5 protein level in chondrocytes measured by western blot.
